# Commencements

**Published:** 1896-06

**Authors:** 


### COMMENCEMENTS.

### Missouri Dental College.

The Dental Department of Washington University—thirty-first annual commencement exercises of the Missouri Dental College were held in St. Louis, Saturday, April 25, 1896. The following graduates received their diplomas:

| NAME. | RESIDENCE. | NAME. | RESIDENCE. |
|---|---|---|---|
| William Bruce Bagbe | Missouri | George Hart Owen | Missouri |
| Harry King Barnett | Illinois | Alonzo William Rue | Illinois |
| Edgar Herbert Bragg | Missouri | Edward Guernsey Simmons | Missouri |
| Peter Thomas Cunningham | Missouri | Elmore Hiram Smith | Missouri |
| Alfred Darst Fuller | Missouri | John Mead Sloan | Missouri |
| Max Fendler | Missouri | Nathaniel Benjamin Stanza | Missouri |
| Harry Moll Fisher | Ohio | Willam Morris Tuttle | Missouri |
| James Fraer Gwinner | California | Walter Edgar Urban | Missouri |
| Charles Joseph Garcia | Missouri | Stephen Huff Voyles | Indiana |
| William Eugene Heatherly | Missouri | August Jos Waldschmidt | Germany |
| Charles Ernst Edw Hesemann | Illinois | Charles Henry Wharton | Iowa |
| Eugene Jordan Hume | Missouri | William Simpson Wallace | Illinois |
| Henry LeGrand Jones | Missouri | Robert Daniel Woelk | Illinois |
| Henry Frank William Koch | Missouri | Florian John Wioser | Illinois |

### University of Maryland—Department of Dental Surgery.

The annual commencement exercises of the Department of Dental Surgery of the University of Maryland were held at the Academy of Music, Baltimore, Md., on Wednesday, April 1, 1896, at 3 P. M.

The address to the graduates was delivered by Rev. Timothy P. Frost, D. D., and the class oration by Harley B. Lindsay, D. D. S.

The number of matriculates was two hundred and four.

The degree of D. D. S. was conferred on the following graduates by Bernard Carter, Esq., Provost of the University:

| NAME. | RESIDENCE. | NAME. | RESIDENCE. |
|---|---|---|---|
| H T Armstrong | New Brunswick | J M Greenough | Jamaica, W I |
| William J Baker | Pennsylvania | Wm H Haller | Virginia |
| Mabrey B Bell | Maryland | Vernon Hiscox | Connecticut |
| John P Best | South Carolina | William D Hopkins | Maryland |
| Cornelius H Bishop | New York | Andrew Huffman | West Virginia |
| Barnet Bliss | Russia | Clarence L Jones | Ohio |
| D L Boozer jr, B S | South Carolina | Antonio S La Rue | Connecticut |
| George L Bressler | Pennsylvania | H B Lindsay, A B | South Carolina |
| George G F Brook | California | T David MacLeod | Nova Scotia |
| Samuel O Brookes | Virginia | Willie J McMinn | North Carolina |
| Marcus Chargin | Russia | Austin H Mowel | New York |
| Robert B Cockrill | Montana | John C B Patrick | South Carolina |
| Robert L Combs | Texas | James W Pierce | Alabama |
| W F Connally | Georgia | Charles H Perdue | Georgia |
| Webster H Cooper | North Carolina | Samuel A Shadrach | Virginia |
| John Sidney Couret | Louisiana | Richard L Simpson | Virginia |
| Howard Eastman | Maryland | Thomas McC Smith | North Carolina |
| John J Ellis | South Carolina | Bennie E Thomas | Texas |
| E J Etheredge | South Carolina | J Cromwell Truby | Pennsylvania |
| Henry Frantz | Illinois | Abraham Weinberg | South Carolina |
| Elias J Friedland | Russia | Albert F Willets | Maine |

### Chicago Northwestern University—Dental Department.

The annual commencement of the Dental Department of the North Western University was held at the Auditorium Recital Hall, Chicago, April 24, 1896, at 2 o'clock, P. M.

The degree of D.D.S. was conferred on the following persons.

| | | |
|---|---|---|
| Maurice P Apmadoc | Charles W Groff | Harry R Rebman |
| Mungo Barr | A J Hockings | J B Simpson |
| Charles J Beers | Harry H Howard | E S Tebbetts |
| Isaac Burton | Adolph F Kortebein | Fredus A Thurston |
| C A Cheney | Frank T Long | Jose F Valentin |
| Arthur R Church | J E McIntosh | George Y Wilson |
| Arthur M Corbin | Will H McKelvie | Orville W Wilson |
| George P Doolittle | J Frank Moore | Charles H Wise |
| Henry L Garrison | Fred B Noyes | Ernest A Worsdell |
| George Gillespie | H A Potts | C H Wright |

### University of Buffalo—Dental Department.

The fourth annual commencement exercises of the Dental Department of the University of Buffalo was held, in connection with the Departments of Medicine and Pharmacy, in Music Hall, in the city of Buffalo, on the evening of Tuesday, May 5, 1896.

The oral examinations, before the Board of Curators, were held during the day, and the members of the Senior Class, who had passed the Faculty examinations, were unanimously recommended to the Council of the University for the degree of Doctor of Dental Surgery, which was accordingly conferred by the Chancellor.

The number of matriculates for the session was one hundred and eighty-six.

The number of graduates was thirty-six, as follows:

| NAME. | RESIDENCE. | NAME. | RESIDENCE. |
|---|---|---|---|
| Arthur C Bean | New York | Eugene L Martin | New York |
| Edward W Bridgman | New York | Emmet E Mills | Pennsylvania |
| Charles K Buell | New York | Wilhelm Muller | Germany |
| Sidney W Bunting | Canada | Wesley A Parish | New York |
| John H Cameron | New York | James M Raimi | New York |
| Victor A Clapp | New York | Richard B Redwan | New York |
| Edmund T Comstock | New York | Lewis P Sanford | New York |
| Horace S Cutler | New York | Henry F Squire | New York |
| Charles S Decker | New York | Peter A Stadlinger | New York |
| William B Dickson | Canada | Earl S Starkweather | New York |
| Carl S Eaton | New York | Walter S Stevenson | New York |
| Charles E Featherstone | Canada | Charles A Stewart | New York |
| James H Gillam | Canada | Jay G Van Valkenburgh | New York |
| Thomas G Gibson | Canada | Henry D Warren | New York |
| Ralfe M Harlan | California | Harry C Webb | New York |
| Arthur Kidder | New York | Carl J Woodworth | New York |
| Frank G Lugsdin | Canada | Douglas H Young | New York |
| Herbert J Lyle | Canada | Paul B H Zuedenfeldt | Germany |

### Cincinnati Academy of Dentistry.

Some two or three months ago a new Dental Society was organized in Cincinnati with the above caption. It holds its meetings on the fourth Monday evening of each month. At the last meeting the officers were elected for the ensuing year:

President, W. T. McLean, M. D., D. D. S.; Vice-president, A. I. F. Buxbaum, D. D. S., M. D.; Secretary, Wm. Lockman, jr.; Treasurer, J. F. Clayton.

Out of the one hundred and forty dentists in Cincinnati there ought to be two good dental societies. Will not each be a stimulus to the other? It is to be hoped that this will be the result.

### Indiana Dental College.

The seventeenth annual commencement exercises of the Indiana Dental College were held in the Grand Opera House, Indianapolis, Ind., on Tuesday evening, March 24, 1896.

The annual address was delivered by Judge W. P. Fishback.

The number of matriculates for the session was one hundred and fifty-seven.

Dr. G. E. Hunt presented diplomas to the following graduates:

| NAME. | RESIDENCE. | NAME. | RESIDENCE. |
|---|---|---|---|
| B M Ackman | Indiana | E H Kreis | Illinois |
| P D Ballou | Indiana | W M Kunkle | Indiana |
| Jennie H Becker | Indiana | M E Little | Indiana |
| T T Brand | Ohio | H T Locy | Indiana |
| L R Booze | Indiana | H R Martin | Ohio |
| J Q Byram | Indiana | E S Miller | Indiana |
| R M Callaway | Indiana | H B M Nutt | Indiana |
| O C Carr | Michigan | Hiram Porter | Indiana |
| F B Cochran | Indiana | E G Prall | Indiana |
| G R Conover | Indiana | T C Rutledge | Indiana |
| R E Culver | Indiana | W P Shortridge | Indiana |
| B H Edwards | Ohio | M V Smith | Indiana |
| A F Eiteljorg | Indiana | J W Vance | Illinois |
| W M Ellison | Indiana | C C Van Scoyoc | Illinois |
| J F Gant | Indiana | J C Vaughn | Indiana |
| M M Gilbert | Indiana | R S Viberg | Indiana |
| E G Glasgo | Indiana | W E Walker | Ohio |
| J R Harrington | Indiana | H D Weller | Ohio |
| Sara Harris | Indiana | G N Wickwire | Indiana |
| W F Huddle | Indiana | V V Wiliiams | Indiana |
| E S Hunt | Indiana | T J Wilson | Indiana |
| R M John | Indiana | | |

### Baltimore College of Dental Surgery.

The fifty-sixth annual commencement exercises of the Baltimore College of Dental Surgery was held at the Lyceum Theater, Baltimore, Md., on Friday evening, March 20, 1896.

The annual oration was delivered by Rev. Edward H. Robbins, and the valedictory by E. F. Early, of North Carolina.

The number of matriculates for the session was two hundred and five.

The degree of D. D. S. was conferred on the following graduates by Professor M. W. Foster, Dean:

| NAME. | RESIDENCE. | NAME. | RESIDENCE. |
|---|---|---|---|
| F D Ashworth | California | M D King | North Carolina |
| J B Askew | Mississippi | C W Motley | Virginia |
| W C Ames | Virginia | E S McIlvaine | Pennsylvania |
| A R Badgley | Ohio | H W Murry | New Brunswick |
| E R Baker | Pennsylvania | L S McElrath | Virginia |
| J G Broad | Pennsylvania | P J McElrath | West Virginia |
| W W Bruck | Germany | R S Peabody | Connecticut |
| A D Coale | Maryland | W J Rolston | Canada |
| C F Chandler | Alabama | E A Stallings | Alabama |
| L E Chevremont | Porto Rico | F Staton | North Carolina |
| R L Carr | North Carolina | J B Saxby | California |
| F C Day | New York | S L Syckes | Pennsylvania |
| E E Erhart | Florida | T P Sullivan | Massachusetts |
| E F Early | North Carolina | W C Sprague | New Brunswick |
| C H Fallon | New Jersey | C A Thompson | North Carolina |
| E P Fitch | Connecticut | J D Tench | Florida |
| J N Garlinghouse | New York | A C Wall | Hawaii |
| J D Haggerty | New Jersey | C A Whitehead | North Carolina |
| J D Hunter | New Jersey | C M Wells, Ph. G | Maryland |
| M Holmboe | Norway | L K Walz | Virginia |
| C W Kammer, Ph. G | Maryland | W F Wilkinson | New York |
| H E Kelsey | North Carolina | C Zimmerman | Pennsylvania |

### Cleveland University of Dentistry and Surgery—Dental Department.

The annual commencement exercises of the Dental Department of the Cleveland University of Medicine and Surgery were held, in connection with those of the Medical Department, at Association Hall, Cleveland, Ohio, on Tuesday evening, March 24, 1896.

The number of matriculates for the session was twenty-three.

The degree of D. D. S. was conferred on the following graduates:

| NAME. | RESIDENCE. | NAME. | RESIDENCE. |
|---|---|---|---|
| Thomas W Blanton | Ohio | Hoyt M Lance | Ohio |
| Joseph S Ewald | Ohio | George H Ormeroid | Ohio |
| Frank S Hunter | Pennsylvania | Edith N Sloan | Ohio |

### Philadelphia Dental College.

The thirty-third annual commencement exercises of the Philadelphia Dental College were held at the American Academy of Music, Philadelphia, Pa., on Thursday, March 5, 1896, at 12 o'clock, M.

The address to the graduates was delivered by Professor S. B. Howell, M. D., D. D. S., and the valedictory address by Charles N. White, D. D. S.

The number of matriculates was four hundred and nine.

The degree of D. D. S. was conferred on the following graduates by ex-Governor James A. Beaver, President of the Board of Trustees:

| NAME. | RESIDENCE. |
|---|---|
| T B Ackley | Pennsylvania |
| Frank G Baldwin | Connecticut |
| L Charles Bechtle | Pennsylvania |
| M U Boatwright | South Carolina |
| L P Bourgeois | Louisiana |
| B C Brock | Iowa |
| Frederic A Brosius | Germany |
| Arthur E Brown | Canada |
| Wm E Bryce | Canada |
| Ellen R Carr | Connecticut |
| W J Carney | West Virginia |
| Horace P Carty | New Jersey |
| Arthur W Chance | Oregon |
| Charles B Cock | Texas |
| J Fred Colomb | Louisiana |
| Wm M Conn | Canada |
| C S Copeland | New Hampshire |
| T F Cottingham | Delaware |
| S P Courtney | Pennsylvania |
| F E Cravath | New York |
| James A Crawford | Pennsylvania |
| F G Crispin, Ph. G. | Pennsylvania |
| Wm H Cummings | Massachusetts |
| Thomas M Daniels | Massachusetts |
| Otto O Davis | Pennsylvania |
| A B Davis | Massachusetts |
| P P Dorr | Iowa |
| John J Dwyer | Massachusetts |
| Charles E Ellegood | Massachusetts |
| John P Erwin | New Jersey |
| F H Espenschade | Pennsylvania |
| J Kurtz Eyler | Pennsylvania |
| Byron M Fell | Pennsylvania |
| James A Finnerty | Pennsylvania |
| George N Fry | Pennsylvania |
| W P Galloway | Iowa |
| W A Gamble | Pennsylvania |
| Leon K Glusman | Russia |
| Arthur T Glew | Canada |
| B C Green | Illinois |
| E J Griffin | North Carolina |
| Henry C Guldin | Pennsylvania |
| Ernst K Gyllang | Sweden |
| Ezra Y Harrison | Pennsylvania |
| H R Hauenstein | Mississippi |
| Homer Heberling | Pennsylvania |
| Emil L H Herbst | Germany |
| Ferd Hinckley | Washington |
| Clarence E Holt | Maine |
| J Pedro Horcasitas | Mexico |
| C G Hughes | Pennsylvania |
| L D Johns | Wisconsin |
| H Furman Jones | New Jersey |
| J Edward Jordan | Pennsylvania |
| Wadea Kassab | Syria |
| Wm Kay | Scotland |
| Wm Kiesau | Iowa |
| R De Vere King | Pennsylvania |
| Wm H Kelly | Pennsylvania |
| Wm A Knapp | Connecticut |
| J Oscar Laird | Iowa |
| H H Lamb | Canada |
| W J Leattor | Pennsylvania |
| Harry F Lotz | Illinois |
| Melvin Markley | Nebraska |
| E Leslie Mason | Maine |
| Albert L Miller | Pennsylvania |
| Robert M Miller | Massachusetts |
| Oswald S Mitivier | Massachusetts |
| John E Morgan | Kansas |
| W G Murray | Canada |
| Joseph M Myers | Pennsylvania |
| John F McCue | Massachusetts |
| Thos J McLernon | Pennsylvania |
| J E McMillan | California |
| Richard Nash | British Columbia |
| H L Patterson | Canada |
| Gray B Perl | Texas |
| James L Peak | Missouri |
| B W Percival | Massachusetts |
| James D Price | Pennsylvania |
| Robert S Rendall | California |
| Sidney J Rauh | Ohio |
| Roy R Ridgely | Illinois |
| J S Robertson | Australia |
| Fred L Rounds | Massachusetts |
| C L Russell | Pennsylvania |
| H E Sangster | Canada |
| J Schleppegrell, M D | South Carolina |
| A J Shanacy | Canada |
| T A Shingle | Pennsylvania |
| H S Skidmore | New York |
| A F Slater | Massachusetts |
| Edbert A Smith | New York |
| Frank R Smith | New York |
| George S Smoyer | Pennsylvania |
| Welcom C Snover | Pennsylvania |
| G H Sweet | Canada |
| William J Taylor | Canada |
| O D Trutt | Pennsylvania |
| William S Teller | New York |
| Charles B Twitchell | New York |
| Benjamin L Tyler | Pennsylvania |
| W M Van Atter | Canada |
| G Van Antwerp jr | Alabama |
| W L Van Buskirk | Pennsylvania |
| W W Vanderhoof | New York |
| J A Wark | New York |
| John D White | Washington |
| Charles N White | New York |
| J Van Wilson | Pennsylvania |
| J E Wolverton | New Jersey |

### Vanderbilt University—Dental Department.

The seventeenth annual commencement exercises of the Dental Department of Vanderbilt University were held in the University Chapel on the evening of March 26, 1896, the Chancellor of the University, J. H. Kirkland, presiding.

Dr. W. H. Morgan, Dean of the Department, delivered the charge to the class, and the valedictory was delivered by E. F. Comegys, of Texas.

The number of matriculates was one hundred and fifty-one.

The number of graduates was twenty-eight.

The degree of D. D. S. was conferred by the Chancellor upon the following named-persons:

| NAME. | RESIDENCE. |
|---|---|
| George C Albright | South Carolina |
| Arthur Barnett | Alabama |
| John Henry Boozer | Texas |
| Moscow B Cortler | Tennessee |
| Edward F Comegys | Texas |
| James Patterson Day | Florida |
| Alfred Wm Dupuy | Alabama |
| John Cecil Felix | Kentucky |
| Charles G Foulks | Alabama |
| James Blount Jordan | Tennessee |
| Wm F McKenney | Indiana |
| Joseph T Meadors | Tennessee |
| Thomas F Nanny | Kentucky |
| George W Parker | Ireland |
| Robert L Parker | South Carolina |
| James Fogg Pickens | North Carolina |
| Louis Frederic Riggs | Canada |
| Edgar Dunn Rose | Texas |
| Robert G Rothrock | Tennessee |
| Jesse Taylor Spain | Tennessee |
| George A Sladen | |
| Wm Andrew Taylor | North Carolina |
| John C Whitefield | South Carolina |
| Byrne A Wilson | Missouri |
| Albert B Wiggonton | Illinois |
| Charles B Woodard | Tennessee |
| Lucian T Wyllis | Tennessee |

### Birmingham Dental College.

The third annual commencement exercises of the Birmingham Dental College were held at O'Brien's Opera House, Birmingham, Alabama, on Friday evening, April 3, 1896.

The address to the graduates was delivered by Dr. J. H. Phillips, Superintendent of Education of the city of Birmingham, and the valedictory address by William B. Fulton, D.D.S., of the graduating class.

The number of matriculates for the session was forty-two.

The degree of D. D. S. was conferred on the following graduates by T. M. Allen, D. D. S., Dean:

| NAME. | RESIDENCE. |
|---|---|
| Robert E Chafer | Florida |
| W H Jett | Alabama |
| L A Crumly, B S | Alabama |
| G Mansfield Lathem | Alabama |
| W B Fulton, B S | Alabama |
| Arthur D Wright | Alabama |

### Pennsylvania College of Dental Surgery.

The fortieth annual commencement exercises of the Pennsylvania College of Dental Surgery were held at the American Academy of Music, Philadelphia, Pa., on Thursday evening, April 2, 1896.

The address to the graduates was delivered by Professor C. N. Pierce.

The number of matriculates for the session was three hundred and forty-two.

The degree of D. D. S. was conferred on the following graduates by I. Minis Hays, M. D., President of the College:

| NAME. | RESIDENCE. |
|---|---|
| Francesco Ardella | U. S. of Colombia |
| Rafael Alvarez | U. S. of Colombia |
| R B Anderson | Pennsylvania |
| W E Askin | Pennsylvania |
| Eugene C Baughn | Pennsylvania |
| J E Beatty | Pennsylvania |
| M Y Belber | Pennsylvania |
| Leon Bernstein | France |
| L H Berger | Pennsylvania |
| Mary E Blake | Massachusetts |
| L L Bosianu | Roumania |
| John A Bothwell | Canada |
| C A Bolton | Pennsylvania |
| George J Brown | England |
| George S Bryson | Massachusetts |
| H P Braham | Pennsylvania |
| J D Burley | New Hampshire |
| N P Bugbee | Vermont |
| J W Carter | Pennsylvania |
| E D'Arcy Cannon | Chili |
| Howard Clymer | Delaware |
| Fannie Crouch | Pennsylvania |
| T G Crymes | South Carolina |
| J L Day | Canada |
| W E Darling | Connecticut |
| J H Drexler | Pennsylvania |
| R J Eccles | Pennsylvania |
| Wm Ehni | Connecticut |
| W J Elder | Ohio |
| Mark J Emlin | New York |
| W A Eynon | Pennsylvania |
| Percy Farrar | Connecticut |
| Albert Frank | Pennsylvania |
| Angela Folkmann | Austria |
| Albert Funke | Pennsylvania |
| F W Glass | Pennsylvania |
| T A Hart | Canada |
| B A Hart | Ohio |
| H S Haslett | Pennsylvania |
| Charles F Healy | Vermont |
| Clark V Hile | Pennsylvania |
| H J Horner | Pennsylvania |
| W A Horner | Pennsylvania |
| A Tate Hoffman | Pennsylvania |
| T C Hosterman | Pennsylvania |
| George F Hummel | Pennsylvania |
| John Hurdis | New York |
| E E Huber | Pennsylvania |
| G F Huntingdon | Canada |
| W K Ingram | Nicaragua |
| W A Jaquette | New Jersey |
| Hedwig Kette | Germany |
| L F Keating | New York |
| E W Klingensmith | Pennsylvania |
| Alfred Krouse | Nicaragua |
| S H Langstroth | Canada |
| Rosa Liefshitz | Russia |
| George H Martin | Massachusetts |
| George C Mathison | Canada |
| W B Maratta | Pennsylvania |
| Wm McIntosh | Canada |
| W P McElroy, M D | Pennsylvania |
| D McAlister | Pennsylvania |
| F S McGannon | Canada |
| S McMurray | Pennsylvania |
| J S Mullin | Pennsylvania |
| H R Nehrbass | Wisconsin |
| H N Osler | Pennsylvania |
| J B Pardoe | New Jersey |
| Carlos J Peralta | Costa Rica |
| W Peters, M D., D D S | Germany |
| Herman T Plass | Pennsylvania |
| M Rebecca Rauch | Pennsylvania |
| G C Roder | Louisiana |
| G A Roberts | Canada |
| A P Rogers | Canada |
| C C Robinson | New York |
| R A Rohrer | Pennsylvania |
| F P Rutherford, Ph. G. | Pennsylvania |
| S V Santz | Pennsylvania |
| C D Scholl | Pennsylvania |
| John A Scott | New York |
| E T Sherman | Massachusetts |
| Charles N Smith | Pennsylvania |
| R H Speare | Canada |
| D G Stewart | Pennsylvania |
| E A Theller | California |
| W M Thompson | New Jersey |
| C M Tocornal, M D | Chili |
| E M Treat | Pennsylvania |
| C F Vaughn | New Jersey |
| F F Westwood | New Jersey |
| W Whitelaw | Canada |
| T T Wilkeson | North Carolina |
| W J Willoughby | Canada |
| J H Worthen | New Hampshire |

### University of Iowa—Dental Department.

The fourteenth annual commencement exercises of the Dental Department of the State University of Iowa were held at the Opera House, Iowa City, Iowa, on Monday evening, March 9, 1896.

The annual address was delivered by Professor J. R. Guthrie, A. M., M. D.

The number of matriculates for the session was two hundred and eighteen.

The degree of D. D. S. was conferred on the following graduates by the President of the University:

| NAME. | RESIDENCE. | NAME. | RESIDENCE. |
|---|---|---|---|
| Marshall B Ayers | Iowa | William A Meis | Iowa |
| Sohn P Brennan | North Dakota | Leah Mills | Illinois |
| Clarence P Bevan | Iowa | Berton A Miller | Iowa |
| William H Batcher | Iowa | Otis M Newman | Nebraska |
| Leon H Branson | Iowa | Leon L Poston | Iowa |
| Besse S Casebeer | Iowa | Claude O Pingrey | Iowa |
| Peter F Dempsey | Iowa | Fred A Rowe | Iowa |
| George W Eshelman | Iowa | William A Reque | Minnesota |
| Thomas G Ferreby | Iowa | Ray E Sharp | Wisconsin |
| Samuel F Heverly | Iowa | Walter Stanford | Pennsylvania |
| Richard E Kidder | Iowa | Wm E Sauls | Nebraska |
| Norval Knight | Iowa | Winfred E Tubbs | Iowa |
| Herbert N Kelley | Iowa | Harry A Tobie | Iowa |
| Ira D Lutz | Illinois | Forest G Webber | Iowa |
| Charles B Lewis | Iowa | Hugo Westhofen | Wisconsin |
| Friend C Leslie | Iowa | Herbert H White | Iowa |
| Leon Mead | Pennsylvania | | |

### Meharry Medical College—Dental Department.

The tenth annual commencement exercises of the Dental Department of Meharry Medical College (Central Tennessee College) were held in Nashville, Tenn., on Tuesday, February 4, 1896.

The valedictory was delivered by Edward D. Bulkley, D.D.S., and the address to the graduating class by Rev. John A. Kumler.

The number of matriculates for the session was eighteen.

The degree of D. D. S. was conferred on the following graduates by President J. Braden:

| NAME. | RESIDENCE. | NAME. | RESIDENCE. |
|---|---|---|---|
| C S Boyd | Tennessee | W T Dinwiddie | Kentucky |
| E D Bulkley | South Carolina | | |

### Kansas City Dental College.

The fourteenth annual commencement exercises of the Kansas City Dental College were held at the Auditorium, Kansas City, Mo., on Tuesday evening, March 31, 1896.

The salutatory was delivered by E. M. Blakey, D. D. S.; the Faculty address by Jabez N. Jackson, A. M., M. D.; the valedictory by F. C. Kingsley, D. D. S., and the annual address by W. A. Quayle, D. D.

The number of matriculates for the session was one hundred and fifty-five.

The degree of D. D. S. was conferred on the following graduates by Alton H. Thompson, D. D. S., President of the Faculty.

| NAME. | RESIDENCE. | NAME. | RESIDENCE. |
|---|---|---|---|
| Ernest W Allison | Kansas | Sidney M Major | Missouri |
| Philip G Brumbaugh | Kansas | Edwin A Morrow | Missouri |
| Edwin L Burner | Kansas | James A Morrow | Missouri |
| Clarence E Crown, B S | Nebraska | Edwin A Morgan | Missouri |
| Louis M Breck | Kansas | Scott R Moore | Missouri |
| Ernest M Blakey | Missouri | Harry B Maxfield | Iowa |
| Daniel B Baker | California | Edward C Morton | Kansas |
| George G Brock | Iowa | Robert L Poplin | California |
| James F Copeland | Kansas | Vernon G Palmer | Iowa |
| Marshall P Davidson | Missouri | Frank J Pribil, jr | Kansas |
| William F Driscoll | California | Frederick F Pound | Missouri |
| Henry V Enloe | Missouri | Ira A Roberts | Kansas |
| Joseph K Griffith, M D | Missouri | Robert T Shaw | Kansas |
| George F Hauser | Wisconsin | David N Smith | Nebraaka |
| Henry C Heady | Missouri | Charles V Shoop | Missouri |
| Daniel P Harford | Missouri | Aretas R Scott | Nebraska |
| Ed M Hiner | Kansas | Frederick C Stote | Kansas |
| William D Hall | Missouri | Earle D Toler | Missouri |
| Charles D Jackson | Nebraska | Jesse W Terwilliger | New York |
| Frederick C Kingsley | Kansas | Edmond J Treyer | California |
| Walter E Kendall | Kansas | Charles H Wikoff | Kansas |
| George P Lux | Kansas | Charles F Watkins | |
| Robert D Lowrey | California | William C Young | Missouri |
| LeRoy W Moore | California | | |

